# Secular Trends in Physical Fitness of Children and Adolescents: A Review of Large-Scale Epidemiological Studies Published after 2006

**DOI:** 10.3390/ijerph17165671

**Published:** 2020-08-05

**Authors:** Tanja Eberhardt, Claudia Niessner, Doris Oriwol, Lydia Buchal, Annette Worth, Klaus Bös

**Affiliations:** 1 Institute of Sports and Sports Science, Karlsruhe Institute of Technology, 76131 Karlsruhe, Germany; doris.oriwol@kit.edu (D.O.); lydia.buchal@hotmail.de (L.B.); klaus.boes@kit.edu (K.B.); 2 Institute of Physical Education and Sports, University of Education Karlsruhe, 76133 Karlsruhe, Germany; annette.worth@ph-karlsruhe.de

**Keywords:** motor performance, trend, youth, review, change

## Abstract

Physical fitness (PF) of children and adolescents is an important resource for their future health. Population-based studies, however, rarely report secular changes of PF, although monitoring of these is crucial to deriving information for adequate interventions. This review aims to report trends in PF of children and adolescents. A literature search was conducted in PubMed in July 2019. Cohort studies published in English allowing statements to be made on trends in PF by comparing youth between the ages of four and 18 years were included. The review identified 24 studies from 16 countries meeting the inclusion criteria, with an overall sample size of more than 860,000 children and adolescents. Through a standardized quality assessment tool, we classified two studies as strong, 21 as moderate, and only one as weak. We analyzed specific secular trends separately for the five different dimensions: endurance, strength, speed, flexibility, and coordination. The majority of studies report a decline of PF over time; however, a few studies report conflicting results. Performance in endurance, strength, and flexibility decreased over time, whereas there was no consistent trend reported for speed and coordination. Overall, there is no international standard on examining and reporting changes or secular trends in PF of children and adolescents, and comparability of studies is limited due to heterogeneous conditions of conducting and analyzing PF tests. Consequently, standardized and consistent international monitoring should be implemented.

## 1. Introduction

Monitoring of physical fitness (PF) in youth is important, because PF is known as one of the most relevant resources for health [1,2,3,4] and is regarded as one of the foundations of an active lifestyle [5].

Childhood is a critical period for the development of PF as it lays the foundation for later PF. Young children acquire a wide range of locomotor and object control skills that enable them to learn adaptive, skilled actions and to adjust them flexibly in different contexts. PF is the base on which children can build more specific motor skills or develop movement patterns. The development of motor performance either encourages or discourages an individual to engage in physical activity through limiting one’s opportunities [5,6,7,8,9,10].

Various definitions of the term PF exist. In Germany, the most commonly used definition was published by Bös [11]. According to this definition, five main dimensions of PF can be distinguished: endurance, strength, speed, and coordination, with flexibility as an additional passive dimension [12]. This definition is based on the concept of physical fitness by Caspersen, Powell, and Christenson [13] and is the basis of this analysis.

In many parts of life, systematic monitoring is used to document changes in society and to describe their course over a certain period of time. Considering the high impact of PF on health in childhood and adolescence, it is crucial to examine how PF has changed over time in children and adolescents. Systematic monitoring of PF is thus needed to assess and design interventions and programs aiming to maintain or increase PF [14,15,16,17].

The majority of large, international studies report the current status and development of PF on the basis of independent, aggregated data sets from differently composed cross-sectional samples. Only few studies exist that were designed to investigate secular trends in PF of children and adolescents with a sufficiently large sample size, an investigation period with several measuring points, and a uniform methodology over years. Unfortunately, population-based studies on trends in PF are rare due to the high time and financial burden needed for carrying out the PF tests [18]. Reliable statements on the current status and on changes, such as secular trends, in PF of children and adolescents on population level are therefore rarely reported on a national and international level.

A few review studies reported decreases in PF levels in the last three decades. For example, a review by Bös [19] analyzed secular trends in PF from 1975 to 2000 in different countries using representative data of over 100,000 children and adolescents. A significant decline of PF of −10% was found for this period and results were particularly conspicuous for endurance and flexibility [19]. An extension of this review to cover additional data published until 2006 confirmed these prior observations and additionally, revealed that the decline in PF was lower for children than for adolescents [20]. A summary of international literature on aerobic and anaerobic performance of children and adolescents by Tomkinson [21] included data from over 25 million children and adolescents aged 6–19 years from 27 different countries between 1958 and 2003 [21,22]. For aerobic performance as measured by different field running tests, the same trends were observed as in the review by Bös et al. [20] namely, that aerobic performance declined at an average rate of −0.36% per year during this period [22]. This decrease was particularly evident for aerobic performance after 1970, following a slight increase from 1958 until 1970 [15]. The results for anaerobic performance were different. PF tests for strength and speed showed a general annual improvement of 0.03% (strength) and 0.04% (speed), particularly before 1985. After this period, changes stabilized or declined [21].

To our knowledge, there is no review published after 2006 that includes data on secular trends in PF over all dimensions. Therefore, the aim of this review was to conduct a literature search on secular trends in PF of children and adolescents in large-scale epidemiological studies published since 2006. In addition, we aimed at considering a wide range of different dimensions of PF, including endurance, strength, speed, flexibility, and coordination. The different assessment periods, test procedures, and potential gender effects on PF secular trends were of particular interest. Based on current results of individual studies [14], we hypothesized that the evidence of the published studies on PF in children and adolescents reveals a stabilization on a rather low level and that the decline in PF is no longer as pronounced as noted in the reviews described above.

## 2. Materials and Methods

This review was carried out following the Preferred Reporting Items for Systematic Reviews and Meta-Analysis (PRISMA) statement guidelines [23]. However, registration of this review to record the process using a database such as PROSPERO (International Prospective Register of Systematic Reviews) was not made. See Table S2 Review protocol for a detailed explanation of the methodological options of the researchers, as well as the sequence and procedures to be implemented in conducting this review.

### 2.1. Eligibility Criteria

Studies meeting the following inclusion criteria regarding the PICOS (participants, interventions, comparators, outcomes, and study design) approach [23] were included in this review:

- Participants between the ages of four to 18 years;
- No intervention;
- Comparison with former measurement point(s);
- Statements of trends in the different dimensions of PF;
- Cohort studies with at least two different measurement points;
- Size of study population *n* > 100.

Only studies published in peer-reviewed journals and written in English were considered. Exclusion criteria were study populations characterized by a physical disease or conducted in a clinical setting, such as diabetes or preterm birth. Additionally, studies among participants with a competitive athletic background and studies analyzing relations, effects, or influences of a specific variable such as sociodemographic status were excluded.

### 2.2. Search

The database PubMed was searched for original studies published between January 2006 and July 2019. The search was conducted in July 2019. The search term consisted of three specific sections, including related terms:

- Section 1—Study population: children OR adolescent* OR youth OR child OR Kinder OR Jugendliche;
- Section 2—Study design: cohort stud* OR Kohortenstudie* OR survey OR longitudinal OR trend* OR secular OR follow-up;
- Section 3—Physical fitness: “Motor performance” OR “motorische Leistungsfähigkeit” OR “physical fitness” OR fitness.

Within the three specific sections, at least one term had to be met. The sections as a whole were connected with the AND operator. See Table S3 full research strategy for detailed documentation.

### 2.3. Study Selection

The search was conducted stepwise by two independent reviewers. The data were managed with Citavi 6.3 (Swiss Academic Software GmbH, Wädenswil, Switzerland). In the first selection step, a screening of titles was carried out. In the next step, abstracts were screened for eligibility. Abstracts meeting the criteria were further examined by reading the full text articles. Full texts were also read for studies that had abstracts providing insufficient information about eligibility. Potential studies for inclusion in the review were scanned by at least two reviewers. Disagreements regarding inclusion were solved by discussion. Also, a third independent opinion of a reviewer was considered. Consensus was then achieved in 100% of the cases.

### 2.4. Data Extraction

The descriptive data of each included study were included in an item extraction form by two reviewers and differences solved by discussion. The item extraction contained source (authors, year of publication), sample (sample size, period of testing, country, age), measurements, and PF test items allocated to the different dimensions of PF, relevant for secular trends.

### 2.5. Data Treatment

To determine secular trends in PF of children and adolescents, only relevant results of the studies included were extracted, including changes and differences for the specific test performances between at least two or more measurement points. Findings from studies were further divided into subsamples based on PF test items and by sex. For example, a study which reported two different test items for strength, i.e., sit-ups and standing broad jump, and one test item for endurance, i.e., shuttle-run, each for boys and girls, led to six different subsamples which we analyzed as part of this review. We chose this procedure as it appeared useful to report results in a more specific and detailed way, especially with regard to different PF trends within a given sample. After building subsamples, we analyzed the reported findings and categorized them into three possible trends, i.e., increase, stagnation, and decline. Nonsignificant changes were considered as stagnation. There were no general cut-offs or categories for decrease or increase. We did not analyze the raw data in the present review, but assessed the available results as a decrease or increase if the authors judged the results to be statistically relevant, based on their findings for the respective study.

### 2.6. Quality Assessment

Quality of the studies included was assessed independently by two reviewers using the Effective Public Health Practice Project (EPHPP) assessment tool. The EPHPP assessment tool is a standardized method used for quantitative studies in public health research. This tool incorporates selection bias across participants, study design, confounders, blinding of researchers and participants, data collection methods, withdrawals, and drop-outs in a global quality rating that differentiates between weak, moderate, and strong. Quality was considered strong when none of the items was graded as weak, moderate quality was considered with one weak rating, and weak quality was the result of two or more weak ratings [24].

## 3. Results

**Figure 1:**
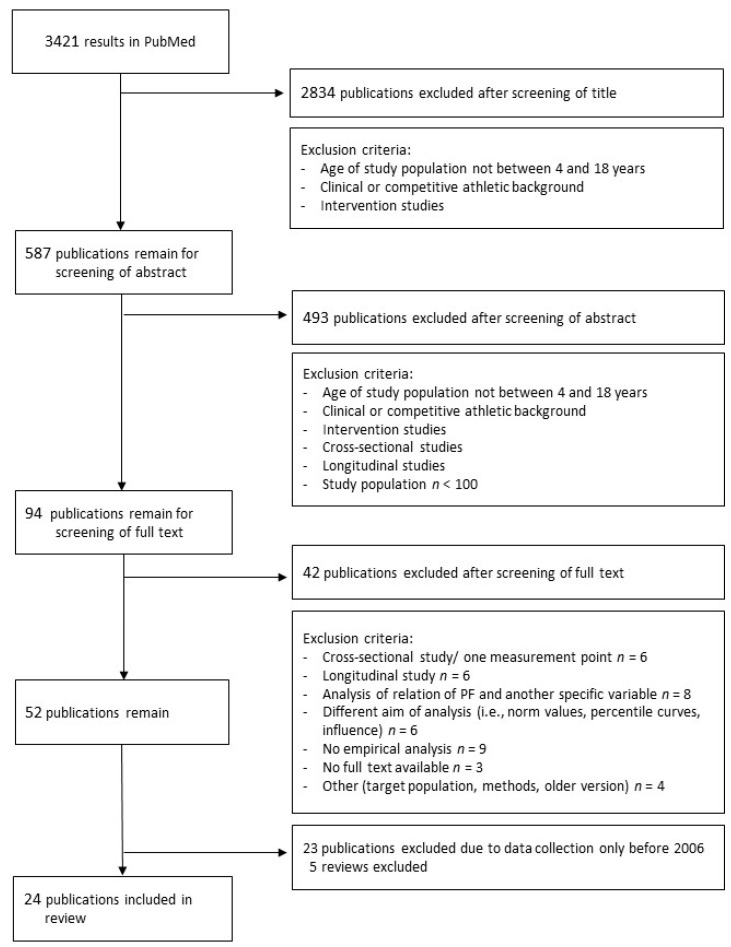
Flow chart of study selection process.

A total of 3421 studies were identified during the initial PubMed search. After the screening titles stage, 587 studies remained. Abstract screening yielded 94 full text articles assessed for eligibility. Twenty-four studies were included in the review for final analysis. Please refer to Figure 1 for a flow chart of the study selection process.

### 3.1. Study Characteristics

Sixteen studies were conducted in Europe [25,26,27,28,29,30,31,32,33,34,35,36,37,38,39,40], three studies in North America [18,41,42], two studies in Australia [43,44], one in China [45], one in Mozambique [46], and one in Brazil [47]. The 24 studies included were published between 2010 [18,30,33] and 2019 [35,43,45], and seven of them had at least two measurement points [18,26,30,31,32,43,44]. The earliest data used in the studies were collected in 1969 [28] and the latest in 2015 [38,43,44]. Sample sizes ranged from 435 [42] to 651,582 [39], and a total of over 860,000 children and adolescents were examined in the studies. The participants were eleven-years old on average, with ages ranging between 3 to 18 years. Sex was equally distributed. In all studies, male and female subjects participated. Study characteristics are summarized in detail in Table 1.

### 3.2. Study Quality

Two studies were classified as strong [28,31], 21 studies as moderate [18,25,26,27,29,30,31,33,34,35,36,37,38,39,40,41,42,43,44,45,46,47], and one study as weak [32]. Due to lacking documentation of withdrawals and drop-outs, most of the studies received a weak rating for this item. This leads to a lower overall rating of these studies. In all studies, data collection methods were considered to be valid and reliable. Regarding an assessment by different dimensions of PF, validity of physical condition tests seems to be higher than that of motor coordination tests (see section Discussion). For this reason, all studies reached a strong rating for this item, reflecting a high-quality main outcome measurement of PF. For PF measurement three relevant confounders were identified, which should be considered in the statistical analysis and were also considered in a separate quality item. If BMI, age, and gender were controlled, we state 80–100% agreement and if only age and gender were controlled, 60–79% agreement was reached for the question of control for relevant confounder. In every study at least some of the relevant confounders were considered. See Table S1 (EPHPP Assessment for included studies) for detailed information on the rating of each study.

### 3.3. Trends in Physical Fitness

To assess secular trends in the PF of children and adolescents, 15 of the 24 studies included carried out test items for endurance [25,28,29,30,31,34,37,38,39,40,41,42,45,46,47]. Fifteen studies used test items for strength [17,27,28,31,33,34,36,37,39,40,41,44,45,46,47], eight for speed [27,30,32,38,39,40,45,46], five for flexibility [18,27,37,40,46], and three for coordination [33,38,40]. A total of 22 different specific test items for PF were carried out in the included studies, but not all were used for analysis. Studies assessing endurance mostly utilized the 20 m shuttle run test [25,30,31,34,37,39,40,41] and the 10 × 50 m shuttle run test [45]. They also used time-related (6 min run [38]), as well as distance-related endurance tests (1 mile run [46], 3000 m run [28]). Three studies included submaximal ergometer testing [29,47] or a treadmill test following the Bruce protocol [42]. Studies assessing strength covered specific test items for different body extremities and the trunk. For the upper limbs and the trunk, handgrip [18,26,30,35,40,46], bent-arm hang [26,30,35,40], ball throw [36,39], and sit-up tests [26,27,32,35,37,40,45] were accomplished. To assess strength of the lower limbs, the standing broad jump test [26,27,30,32,33,35,36,40,43,44,45] or a vertical jump test [39] was carried out. For assessing speed, sprints over a short distance (20 m [38], 30 m [39], 40 m [27], 50 m [45]) or short shuttle run tests, such as the 4×10 m shuttle run [30,32] or the 5×10 m shuttle run [40,46], were made. All studies assessing flexibility used the sit-and-reach test [18,27,37,40,46]. Assessment of coordinative abilities included an obstacle course, target throwing, or balancing backwards [33,38,40]. For an overview of all test items used in the studies to assess the different dimensions of PF, see Table 1.

Within the 24 studies included, there were 148 subsamples: 35 subsamples reported an increase of PF, 20 subsamples showed stagnation with no significant changes over the period analyzed, and 93 subsamples found a negative trend in different PF tests. Results of trends in PF for the specific dimensions are presented in Table 2.

#### 3.3.1. Endurance

Fifteen studies include test items for endurance, which resulted in 36 subsamples. Seven subsamples showed an increase in endurance performance, which was assessed with the 20 m shuttle run test [30,37,41,42]. For example, comparison of studies conducted in Spain in 2001 and 2007 show that participants reached higher stages in the shuttle run test, indicating better endurance at the later time point. In 2001, boys aged between 12.5 and 17.5 reached 6.7 stages ± 2.4 and in 2007 7.7 stages ± 2.4 (Cohens *d* = 0.41; *p* = 0.001) [30]. The performance in the 20 m shuttle run test of 6 to 7-year-old children in Greece between 1992 and 2007 differed by 21% for boys and 26% for girls [37].

Stagnation of endurance performance was observed in six subsamples [31,38,41]. For example, Palomäki et al. [31] found no significant changes in the sample of Finnish adolescents between 2003 and 2010.

The majority of studies (i.e., 23 subsamples) suggested a decline in endurance performance in children and adolescents [25,28,29,34,38,39,40,42,45,46,47]. In a Norwegian cohort study, 3000 m running time increased by 10% for boys and 6% for girls over three decades [28]. There was, however, a large variability in the amount of decline in the 20 m shuttle run test. Sandercock et al. [34] found an annual decline of 0.7% for boys and 0.9% for girls between 1998 and 2008 in 10 to 11-year-old children from England and the updated data of 2014 confirmed this upward trend. Lithuanian schoolchildren achieved almost twice as high stages in the 20 m shuttle run test in 1992 compared to the latest measurement in 2012. However, the decline became smaller between 2002 and 2012 [40]. A stratification of performance in quartiles according to BMI-categories showed that the percentages of both genders in the quartile of excellent performance decreased substantially, while the poor percentage in the poor performance quartile increased within all BMI-categories. For example, the percentage of normal-weight boys in the excellent quartile was 36.3% in 1997 and decreased to 21.1% in 2007. In turn, the percentage changes for the poor quartile rose from 21.8% in 1997 to 32.1% in 2007 [39].

The mean annual decline reported by Boddy et al. [25], who conducted twelve different measurements between 1998 and 2010, was 1.34% for boys and 2.29% for girls.

#### 3.3.2. Strength

For lower limb and leg strength, 13 studies with 34 subsamples were identified and most of them utilized standing broad jump [26,27,30,32,33,35,36,37,40,43,44,45]. Six subsamples found a trend towards increased performance [26,33,37]. Greek boys aged 6 to 7 years jumped 0.36 m farther in the 2006/2007 cohort compared to 1992/1993 [37]. The difference in 10 to 11-year-old children from the UK was approximately 0.07 m between 1998 and 2008 [26], and German children also significantly increased their standing broad jump performance between 1989 and 2007 [33]. Two studies with four subsamples found no significant changes during a time period of ten years [27,39]. Also, Roth et al. [33] reported no change in performance. Twenty-two subsamples, therefore most of the studies, suggested a decreasing trend for the lower limb strength in children and adolescents [30,32,33,35,36,40,43,44,45]. For Australian children aged 11 to 12 years, the mean difference between 1985 and 2015 was 0.11 m [43], and 9 to 15-year-old children jumped approximately 0.07 m shorter in the later measurement point [44]. Moliner-Urdiales et al. [30] reported a decrease of 0.09 m for boys and 0.12 m for girls from 2001 to 2007 in Spain. Other European studies also found a negative trend [32,35,36,40]. In contrast to this, China first reported an increase in standing broad jump performance until 1995, which was followed by a decline until the last measurement in 2014 [45].

For upper limb strength and more specifically, arm strength, the bent-arm hang test was performed in four studies with eight subsamples [26,30,35,40]. None of the studies reported an increasing trend of performance. Between 2001 and 2007, hang time was similar and no changes were found in Spanish adolescents [30]. Apart from this stagnation, three studies with six subgroups reported a decline in upper body strength [26,35,40]. In the study of Cohen et al. [26] on 10-year-old children, significant annual declines by 1.27% for boys and 2.27% for girls were shown. In the extension of Sandercock et al. [35] with a third measurement conducted in 2014, this trend continued. Over the 20 years that were analyzed, the decline in hanging time of Lithuanian children was most pronounced during the last decade, from 2002 to 2012 [40].

In five studies with ten subsamples, the handgrip test was assessed [18,26,30,35,46]. Dos Santos et al. [46] reported an increase for boys from 20.11 kg to 20.95 kg and 22.51 kg over the years 1992, 1999, and 2012. For girls, however, a decline was observed. The negative trend was also found in nine of the ten subsamples. Canadian children showed a significant negative change over all examined age groups from 6 to 19 years between 1981 and 2009. Handgrip strength in boys aged 11 to 14 years was 6 kg lower in the second cohort and in 15 to 19-year-old girls handgrip strength decreased from 60 kg to 54 kg [18]. The shorter time period from 2001 to 2007 revealed a significant decrease of 4.5 kg for Spanish adolescents [30]. The most recent measurement in 2014 for 10-year-old English girls also revealed lower handgrip in 2014 compared to 2008 and 1998 [35].

Sit-ups were utilized to measure trunk strength in seven different studies and 18 subsamples [26,27,32,35,37,40,45]. Greek and Portuguese children improved their performance from 1993 to 2013 [27] and 1992 to 2007, respectively [37]. The 6 to 7-year-old boys performed 4.8 and girls 5.2 more repetitions at the second measurement [37]. One of the subsamples, however, displayed a stagnation of trunk strength [40], while four studies with ten subsamples showed a decline [26,32,35,45]. For example, sit-up performance in English children declined by 27% from 1998 to 2008 and by an additional 19% from 2008 to 2014 [26,35]. Earlier measurements in a Chinese sample showed an initial increase from 1985 until 2000, which was subsequently followed by a decline in the following five years [45].

#### 3.3.3. Speed

Eight of the studies included 20 subsamples focused on speed performance of children and adolescents [27,30,32,38,39,40,45,46]. Five studies reported an increase of speed over time in eight subsamples [27,30,38,39,45]. When the 30 m sprint of Greek 8 to 9-year-old children was stratified into quartiles, the percentage of normal-weight girls in the excellent quartile increased from 28.6% in 1997 to 34.9% in 2007 [39]. Likewise, Spanish adolescents aged 12.5 to 17.5 years improved their speed between 2001 and 2007 [30], and German 6-year old children showed a mean improvement of 2.7% (boys) and 4.3% (girls) per decade [38].

Three subsamples, by contrast, reported a stagnation of speed [27,40]. Even though Venckunas et al. [40] found an improvement for boys between 1992 and 2002, no change was shown from 2002 to 2012 and there was no change in girls for the entire study period. In addition, Portuguese girls did not exhibit any significant change over 30 years [27].

Three studies with nine subsamples reported a decline in sprint performance [32,38,45,46]. Chinese girls at an age of 12 became slower over the measurements from 1985 until 2014 [45]. In 1992, Mozambican 8 to 15-year-old boys needed 21.88 s to finish the 5 × 10 m shuttle run test; in 2012, they needed 22.51 s. The same trend was observed for girls, who needed 22.49 s in 1992 and 23.66 s in 2012 [46].

#### 3.3.4. Flexibility

All five studies with 12 subsamples measured flexibility with the sit-and-reach test [18,27,37,40,46]. One study from Greece reported a positive secular trend, i.e., Smpokos et al. [37] observed an increase of 22% for boys and 13% for girls between 1992 and 2007.

In contrast to this, the other four studies reported a decrease in flexibility over time [18,27,40,46]. Mozambican 8 to 15-year old children were less flexible in 2012 and 2002 than in 1992. For example, performance of girls decreased from 38.03 cm in 1992 to 36.52 cm in 2002 to 35.41 cm in 2012 [46]. A similar trend of decreasing flexibility was observed for Lithuanian children [40]. In line with this, Tremblay et al. [18] analyzed trends of performance in the sit-and-reach test of Canadian children and adolescents, and reported lower flexibility in 2009 compared to 1981 across all age groups; i.e., performances of the 15 to 19-year-old boys decreased significantly from 30 cm to 24 cm.

#### 3.3.5. Coordination

Three studies were found to assess coordination, which resulted in 10 subsamples using various tests [33,38,40]. Spengler et al. [38] found a relatively strong positive trend in the static stand test for German 6-year-old children with values increasing by 22.8% (boys) and 41.1% (girls) between 2006 and 2015. Similarly, balance performance of Lithuanian children and adolescents aged 11 to 18 years improved from 1992 to 2012 [40]. This effect was observed for all age groups but was more pronounced for girls than for boys. Roth et al. [33] reported no change in coordination performance between 1989 and 2007. However, the authors reported a decline for 3 to 6-year-old children in balancing backwards and target-throwing tasks between 1985 and 2007 [33].

## 4. Discussion

The aim of this review was to conduct a literature review of secular trends in PF of children and adolescents in large-scale epidemiological studies, which have at least one measurement in or after 2006.

Within the 24 studies included, there were 148 subsamples: 35 subsamples (24%) reported an increase of PF, 20 (13%) a stagnation with no significant changes over the analyzed period, and 93 subsamples (63%) found a negative trend in different PF test items.

For endurance, 23 subsamples showed a declining trend, seven subsamples an increase, and six subsamples a stagnation. The declining trend is especially obvious in studies with measurement times that are older and with long time periods between follow-ups [42,45,47]. The same findings were supported with the latest study by Greier et al. [16], who analyzed Tyrolean boys aged 10 to 14 years. Over the measurement time points in 1972 and 2015, endurance declined by 15% [16]. In addition, for Chinese children and adolescents, Bi et al. [48] showed a decline between the latest measurement points of 1995 and 2014, and a stagnation from 1985 until 1995. Increase and stagnation of endurance were mostly found in studies covering measurements after 2000 [30,31,40]. These findings are partly in line with a recent review of Tomkinson et al. [49] that covered 137 studies with measurement periods from 1981 until 2014. Cardiorespiratory fitness decreased in the 1980s and 1990s, especially, but with a slowing trend since the 2000s. The decline then stabilized [49].

For strength, 47 of the 70 subsamples showed a decline. Most of the studies reporting a decline were characterized by long time periods between follow-ups. The findings were highly inconsistent, depending on the part of body for which the strength test was conducted.

This inconsistency within findings for certain parts of the body was also obvious in several other studies. Albon et al. [50] found an increase for performance of sit-ups, but no significant changes for standing broad jump between 1991 and 2003. Tomkinson et al. [21], by contrast, analyzed data of over 20 million young people from 27 countries with measurements between 1958 and 2003 and found a general increase by 0.03% per year for the lower limbs. From the late 1950s until the 1980s, performance in standing broad jump increased consistently and then stabilized before a 15-year decline. Huotari et al. [51] reported no significant changes in standing broad jump for Finnish adolescents, a decline for the bent arm hang, but an increase for sit-ups.

For speed, the 20 subsamples can be divided into eight subsamples showing an increase, three revealing stagnation, and nine exhibiting a decline. For this reason, it is impossible to determine a direction of trend. Other research yielded the same results. Matton et al. [52] reported an increase of performance of Flemish adolescents between 1969 and 2005, while the performance of Estonian and Lithuanian children and adolescents measured in 1992 and 2002 did not change [53]. An overall increase of 0.04% per year was found by Tomkinson et al. [21] when analyzing data measured between 1958 and 2003. Since 1985, however, this increase has stabilized at values close to zero.

For flexibility, a declining trend was found in the studies. In ten of 12 subsamples, the children’s performance in sit-and-reach tests declined.

For coordination, only ten subsamples of the studies included could be analyzed and this less evidence was specified even more through targeting different aspects of coordination tasks. Roth et al. [33] found stagnation and decline for different coordination tests, whereas the two other studies revealed an increase [38,40].

There is still some uncertainty in these findings as the comparisons are based on independent samples and sampling effects cannot be excluded. However, most of the results confirm the results of previous reviews [19,20,49] and are therefore consistent. It is crucial that PF components are analyzed separately because all components showed different patterns of secular trends over time and demonstrate the essential need for a detailed assessment of trends. The negative trends especially in endurance are alarming, although they slowed down in recent years. Cardiorespiratory fitness represents a key to health in later life. In particular, it is important to keep the absolute level of performance in mind when considering trends, whether positive or negative. Even slight positive changes may only reduce the negative relative change in recent years and, thus, only lead to “stagnation of PF on a low level.”

Given the proven correlation between PF and physical and mental health [54], a “stagnation of PF on a low level” of children and adolescents cannot be the target state. To ensure an active and healthy lifestyle, it is crucial to adequately promote PF of children and adolescents. Regular and consistent monitoring with a standardized method should be implemented internationally and supported by important decision-makers. A common monitoring is recommended to design interventions and programs on a reliable database and should be a strategic aim of international sports policy institutions.

To solve the problem of low comparability of the primary studies analyzed for this narrative review (e.g., different methods, definitions, and samples), eResearch infrastructure should be implemented for storing, linking, and reusing the data. Sustainable access to data enables collaborative work and makes research comprehensible and reusable across disciplinary boundaries. For PF test data, the sport scientific eResearch infrastructure MO|RE data was developed, to store, combine, and evaluate data. The research results are made publicly accessible and citable via the platform [55].

The results are even more difficult to compare due to different sample sizes, statistical analyses, measurement points, and methods. In this context, various other aspects, i.e., socioeconomic status, educational system, and geographical regions, should be considered [14,21].

Coordination is considered an important part of the concept of health-oriented fitness [56]. However, the exact recording of coordinative aspects and their correct interpretation, as well as the comparability of different coordination results are difficult.

Firstly, the construct of coordinative abilities is complex and multidimensional; i.e., different coordination tasks can measure different aspects of coordination (e.g., reaction ability, orientation ability).

Secondly, coordination tasks are usually assessed qualitatively, which makes it difficult to determine trends in coordination. Qualitatively assessed test items often are associated with a low reliability. Estimating reliability for characteristics with a reduced level of characteristic expression (e.g., dichotomous characteristics or ordinal characteristics) is problematic, since correlation coefficients are dependent on both variance and difficulty [57]. It is therefore more difficult to maintain quality criteria for coordination than for interval-scale test items (e.g., strength, endurance) [58]. In summary, for the assessment of trends this means that the results for coordination are not comparable with the results of the more reliable interval-scaled physical condition test items, due to the methodological difficulties mentioned above.

A long time between the first and the last measurements seems to lead to more changes over time in PF. Time should not be too long to obtain a reasonable development. It is recommended to measure PF not only in the beginning and at the end of an investigation, but at periodic intervals for regular and continuous monitoring [14]. In Germany, these requirements are met by the MoMo Study, a nationwide large cohort study that examines trends in PF, already with three waves of measurements since 2003 [14].

When assessing the subsamples, some differences are also due to age and gender. The study by Smpokos et al. [37], which found an increase of endurance, strength, and flexibility, disagreed with the overall findings. The study population was 6 to 7-year-olds. Differences due to age were also found in Australian children with a decreasing trend for older children. Increase was found mainly for children below the age of nine [26,33,37,38,39], while subsamples with older children tend to show even smaller values [28,29,31].

All of the studies included analyzed gender-specific differences. Spengler et al. [38] found a decreasing trend in boys’ endurance, but no significant changes for girls. Lithuanian girls increased their performance in sit-ups, while boys did not [40]. Costa et al. [27] reported an increasing performance of boys for 40 m sprint, while the performance of girls did not increase. However, in contrast to Tomkinson et al. [17] for gender-specific differences, no clear direction was found overall. In the specific dimensions of PF, the strikingly similar results like Tomkinson et al. [21] reported for power and speed cannot be concluded. For a detailed assessment of differences between male and female children and adolescents, a deeper statistical investigation must be performed, i.e., with a meta-analysis.

This review summarized data of studies in 16 countries from all continents and therefore estimates international trends of PF. With data from over 860,000 children and adolescents, the sample size is appropriate. This review was conducted following the PRISMA guidelines, making the approach systematic. Also, we developed a research protocol; for each of the included studies a quality assessment was performed to respect the strength of evidence (see Supplementary Material). However, registration of this review to record the process through a database such as PROSPERO was not made. Although there are several reviews examining trends in PF of children and adolescents, it does provide comprehensive work because it does not focus on one dimension of PF or separate aerobic and anaerobic performances. The review summarizes findings in all dimensions and extends to an actual global analysis, with studies having at least one measurement in or after 2006. The method of building subsamples and categorizing them is as detailed and specific as possible without generating a meta-analysis. However, this results in a different number of subsamples, depending on the variance of conducted tests, and leads to a weighting. The more subsamples built for a study, the stronger its weighted result.

Some methodological limitations of this review should be considered. There was only primary study research in one database of scientific evidences used to identify potential studies. Therefore, the review does not claim to be exhaustive, as gray literature or literature not published in English was not considered (e.g., Albrecht et al. [59]: MoMo Study for Germany). The primary studies used different methods and definitions, or there was large heterogeneity in the study population. The studies analyzed data that differed substantially in methods and characteristics of samples and cohorts. The various sample sizes of different studies were not weighted, but influenced the representativeness of each study. Furthermore, the specific dimensions of PF were measured with different test items; sometimes test items even varied for a single dimension (e.g., seven different test items to measure endurance). No in-depth analyses of the primary data of the studies were made to summarize the results quantitatively by using statistical methods and calculated pooled-effect estimates. We assessed the reported trends dependent on the judgment of the authors in the respective studies. A meta-analysis would be desirable to report the relative change per year and to compare outcomes of studies with common metrics. Furthermore, such a meta-analysis allows determination of existing influences and their strength. This will help us find out whether a valid overall picture can still be obtained.

We considered gender, age, and BMI as relevant confounders of PF, but there are more; socioeconomic status and geographical environment influence the trends in PF as well [14].

We did not include existing reviews reporting secular trends. A logical next step should be to conduct an umbrella review.

## 5. Conclusions

In conclusion, there is an overall declining trend when assessing the findings in PF of children and adolescents. However, these findings vary for the specific dimensions of PF and require specified and detailed reporting.

(1) For endurance, strength, and flexibility, the majority of the primary studies report a decline. This trend towards a deterioration appears to weaken in the more recent studies. The major changes in PF are more likely to be reported in work that goes back further in time (1960s until the 1980s)
(2) For speed and coordination, the same number of studies reported a decrease, increase or stagnation.

Gender- and age-specific differences for trends in PF are small, but changes seem to decrease for adolescents compared to children, and vary for gender in some dimensions without any clear direction. A standardized and regular monitoring of trends to design interventions and programs is needed. This monitoring should also include and report potential influencing factors such as gender, age, sociodemographic, and environmental differences. Furthermore, reporting of specific dimensions is indispensable. This monitoring is crucial to support PF as a resource for future health and as a requirement of an active lifestyle.

## Figures and Tables

**Table 1:** Characteristics of included studies.

Reference	Country	Period	Measurements	Age	Sample Size	Dimension of PF	Test Items
Ao et al. [45]	China	1985–2014	1 per year, 7 overall	12	136,566	Endurance, strength, speed	10×50SR; SBJ, SU, 50 m
Bai et al. [41]	USA	2012–2014	1 per year, 3 overall	5–18	6318	Endurance	20SR
Boddy et al. [25]	UK	1998–2010	1 per year, 12 overall	9–10.9	14,247	Endurance	20SR
Cohen et al. [26]	UK	1998–2008	1 per year, 2 overall	10–10.9	624	Strength	SBJ, SU, HG, BAH
Costa et al. [27]	Portugal	1993–2013	1 per year, 4 overall	10–11	1819	Strength, speed, flexibility	SBJ; SU; 40 m; SAR
Dos Santos et al. [46]	Mozambique	1992–2012	1 per year, 3 overall	8–15	3851	Endurance, strength, speed, flexibility	1 mile, HG, 10×5SR, SAR
Dyrstad et al. [28]	Norway	1969–2009	1 per year, over 40 overall	16–18	4006	Endurance	3000 m
Ekblom et al. [29]	Sweden	1987–2007	1 per year, 3 overall	16	1023	Endurance	SME
Fraser et al. [43]	Australia	1985–2015	1 per year, 2 overall	11–12	3732	Strength	SBJ
Hardy et al. [44]	Australia	1985–2015	1 per year, 2 overall	9–15	7081	Strength	SBJ
Moliner- Urdiales et al. [30]	Spain	2001–2007	1 per year, 2 overall	12.5–17.5	791	Endurance, strength, speed	20SR; SBJ, HG, BAH, 4×10SR
Moraes Ferrari et al. [47]	Brazil	1978–2010	1 per year, 4 overall	10–11	1291	Endurance	SME
Morales- Demori et al. [42]	USA	1983–2010	1 per participant	4–18	435	Endurance	BTP
Müllerova et al. [32]	Czech Republic	1987–2013	1 per year, 2 overall	8–13	896	Strength, speed	SBJ, SU, 4×10SR
Palomäki et al. [31]	Finland	2003–2010	1 per year, 2 overall	15–16	3559	Endurance	20SR
Roth et al. [33]	Germany	1973–2007	1 per year, 4 overall	3–6	2293	Strength, coordination	SBJ, Obstacle Course, BB, TT
Sandercock et al. [34]	UK	1998–2014	1 per year, 3 overall	10–11	925	Endurance	20SR
Sandercock et al. [35]	UK	1998–2014	1 per year; 3 overall	10	920	Strength	SBJ, HG, SU, BAH
Sedlak et al. [36]	Czech Republic	1977–2012	1 per year, 6 overall	5–6	3768	Strength	SBJ, BT
Smpokos et al. [37]	Greece	1992–2007	1 per year, 2 overall	6–7	967	Endurance, strength, flexibility	20SR, SBJ, SU, SAR
Spengler et al. [38]	Germany	2006–2015	1 per year, 10 overall	6–7	5001	Endurance, speed, coordination	6 min, 20 m, SLS
Tambalis et al. [39]	Greece	1981–2007	1 per year, 10 overall	8–9	651,582	Endurance, strength, speed	20SR, VJ, BT, 30 m
Tremblay et al. [18]	Canada	1981–2009	1 per year, 2 overall	7–19	7203	Strength, flexibility	HG, SAR
Venckunas et al. [40]	Lithuania	1992–2012	1 per year, 3 overall	11–18	16,199	Endurance, strength, speed, flexibility, coordination	20SR, SBJ, SU, BAH, 10×5SR, SAR, SLS

Abbreviations: BAH = Bent-arm hang; BB = Balancing backwards; BT = Ball throw; BTP = Bruce treadmill protocol; HG = Hand grip; SAR = Sit-and-reach; SBJ = Standing broad jump; SLS = Single leg stand; SME = Submaximal ergometer test; SU = Sit ups; TT = Target throwing; 4 × 10SR = 4 × 10 m shuttle run; 10 × 5SR = 10 × 5 m shuttle run; 10 × 50SR = 10 × 50 m shuttle run; 20SR = 20 m shuttle run; 1 mile = 1 mile run; 3000 m = 3000 m run; 20 m = 20 m run; 30 m = 30 m run; 40 m = 40 m run; 50 m = 50 m run; 6 min = 6 min run.

**Table 2:** Results of trends in PF for the specific dimensions of PF. A significant result is rated as increase or decrease; a nonsignificant result is rated as stagnation.

**Motor Tests for Endurance**	**Trends in Subsamples (Number of Categorized Subsamples)**		
	**Increase**	**Stagnation**	**Decline**
10 × 50 m run			[45] (4)
20 m – shuttle run	[41] (3); [30] (2); [37] (2)	[41] (3); [31] (2)	[25] (2); [34] (2); [39] (2); [40] (2)
1 mile run			[46] (2)
3000 m run			[28] (2)
Submaximal ergometer test			[29] (2); [47] (2)
Bruce treatmill test			[42] (2)
6 min run		[38] (1)	[38] (1)
∑ endurance: 15 studies (36 subsamples)	∑ (7)	∑ (6)	∑ (23)
**Motor tests for strength**	**Trends in subsamples (number of categorized subsamples)**		
	**Increase**	**Stagnation**	**Decline**
Standing broad jump	[26] (2); [33] (2); [37] (2)	[27] (2); [33] (2) [39] (2)	[45] (4); [43] (2); [44] (4); [30] (2); [32] (4); [35] (2); [36] (2); [40] (2)
∑ leg strength: 13 studies (34 subsamples)	∑ (6)	∑ (6)	∑ (22)
Bent-arm hang		[30] (2)	[26] (2); [35] (2); [40] (2)
∑ arm strength: 4 studies (8 subsamples)	∑ (0)	∑ (2)	∑ (6)
Handgrip test	[46] (1)		[26] (2); [46] (1); [30] (2); [35] (2); [18] (2)
∑ grip strength: 5 studies (10 subsamples)	∑ (1)	∑ (0)	∑ (9)
Sit-ups	[45] (2); [27] (2); [37] (2); [40] (1)	[40] (1)	[45] (2); [26] (2); [32] (4); [35] (2)
∑ trunk strength: 7 studies (18 subsamples)	∑ (7)	∑ (1)	∑ (10)
**Motor tests for speed**	**Trends in subsamples (number of categorized subsamples)**		
	**Increase**	**Stagnation**	**Decline**
4×10 m shuttle run	[30] (2)	[40] (2)	[32] (4)
10×5 m shuttle run			[46] (2)
50 m sprint	[45] (1)		[45] (3)
40 m sprint	[27] (1)	[27] (1)	
30 m sprint	[39] (2)		
20 m sprint	[38] (2)		
∑ speed: 8 studies (20 subsamples)	∑ (8)	∑ (3)	∑ (9)
**Motor tests for flexibility**	**Trends in subsamples (number of categorized subsamples)**		
	**Increase**	**Stagnation**	**Decline**
Sit-and-reach	[37] (2)		[27] (2); [46] (2); [18] (4); [40] (2)
∑ flexibility: 5 studies (12 subsamples)	∑ (2)	∑ (0)	∑ (10)
**Motor tests for coordination**	**Trends in subsamples (number of categorized subsamples)**		
	**Increase**	**Stagnation**	**Decline**
Obstacle course		[33] (2)	
Balancing backwards			[33] (2)
Target throwing			[33] (2)
One leg stand	[38] (2); [40] (2)		
∑ coordination: 3 studies (10 subsamples)	∑ (4)	∑ (2)	∑ (4)

∑ = sum of subsamples.

## Supplementary Materials

The following are available online at https://www.mdpi.com/1660-4601/17/16/5671/s1, Table S1: EPHPP Assessment for included studies; Table S2: Review protocol; Table S3: Full research strategy.
