# Supplementary material for: Secular Trends in Physical Fitness of Children and Adolescents: A Review of Large-Scale Epidemiological Studies Published after 2006
Source: Int J Environ Res Public Health. 2020 Aug 5;17(16):5671. doi: 10.3390/ijerph17165671 (PMC7459672; doi:10.3390/ijerph17165671)
Supplement: Supplementary file 1 [file ijerph-17-05671-s001.zip › Supplementary Material/S2_Review protocol.pdf]

Review Protocol

**Title**	Secular Trends in Physical Fitness of Children and Adolescents: Review of Large-scale Epidemiological Studies Published after 2006
**Authors**	Tanja Eberhardt[1], Claudia Niessner[1], Doris Oriwol[1], Lydia Buchal[1], Annette Worth[2] und Klaus Bös[1]<br>[1] Institute of Sports and Sports Science, Karlsruhe Institute of Technology, Karlsruhe, Germany<br>[2] Institute of Physical Education and Sports, University of Education Karlsruhe, Karlsruhe, Germany
**Reviewers**	Tanja Eberhardt, Claudia Albrecht
**Support / Supervisors**	Klaus Bös

1. Background

Monitoring of physical fitness (PF) in youth is important, because PF is known as one of the most relevant resource for health [1–4] and is regarded as one of the foundations of an active lifestyle [5].
Childhood is a critical period for the development of motor PF as it lays the foundation for later PF. Young children acquire a wide range of locomotor and object control skills that enable them to learn adaptive, skilled actions and to adjust them flexibly in different contexts. PF is the base on which children can build more specific motor skills or develop movement patterns [5, 6].
The development of motor perfomanceMP either encourages or discourages an individual to engage in physical activity through limiting one's opportunities [7–10].
Various definitions of the term PF exist. In Germany, the most commonly used definition was published by Bös [11]. According to this definition, five main dimensions of PF can be distinguished: endurance, strength, speed, and coordination, with flexibility as an additional passive dimension [12]. This definition is based on the concept of physical fitness by Caspersen, Powell & Christenson [13] and is the basic of this analyses.
In many parts of life, systematic monitoring is used to document changes in society and to describe their course over a certain period of time. Considering the high impact of PF on health in childhood and adolescence, it is crucial to examine how PF has changed over time in children and adolescents. Systematic monitoring of PF is thus needed to assess, and design interventions and programs aiming to maintain or increase PF [14–16].

2. Objectives

The objective of this review is to summarize the evidence regarding secular trends in physical fitness of children and adolescents in large- scale epidemiological studies published after 2006

3. Inclusion criteria

**Population / participants**	Participants between the ages of 4 to 18 years
**Interest / exposure**	No intervention
**Comparison group**	Comparison(s) with former measurement point(s)
**Outcome of interest**	Statements of trends in the different dimensions of physical fitness (endurance, strength, speed, flexibility and coordination)

**Study design**	Cohort studies with at least two different measurement points
**Other criteria**	Single study articles published in peer-reviewed journals (full-texts)<br>English language
**Exclusion criteria**	• Study populations characterized by a physical disease or conducted in a clinical setting, such as diabetes or preterm birth. Additionally, studies among participants with a competitive athletic background<br>• Study population N <100<br>• Studies analyzing relations, effects or influences of a specific variable such as socio-demographic status<br>• Articles published in any other language than English
**4. Search Methods**	
**Electronic Databases**	The following databases will be searched: PubMed
**5. Methods of the Review**	
**Details of methods**	All identified articles are exported to a reference manager program. In a first step, duplicates will be removed. In the second step, articles will be screened based on title and abstract, and in the third step, based on full-texts. The whole screening process will be conducted by two reviewers independently.<br><br>Two main reviewers (Tanja Eberhardt, Lydia Buchal) will review the studies. Studies will be included based on agreement of two reviewers, a third reviewer will be contacted (Claudia Niessner) for any disagreements.<br>The same approach will be taken for the study quality assessment.
**Quality assessment**	EPHPP Assessment tool
**Data extraction**	Data will be extracted and summarized in a table, including:<br>• Authors and year<br>• Country<br>• Period<br>• Measurements<br>• Age<br>• Sample size<br>• Dimension of physical fitness tested<br>• Test Items
**Data synthesis**	• Categorization into three directions of trends<br>Increase- Stagnation- Decrease

References

1. Hamer M, O'Donovan G, Batty GD, Stamatakis E. Estimated cardiorespiratory fitness in childhood and cardiometabolic health in adulthood: 1970 British Cohort Study. Scand J Med Sci Sports. 2020;30:932–8. doi:10.1111/sms.13637.
2. Ortega FB, Ruiz JR, Castillo MJ, Sjöström M. Physical fitness in childhood and adolescence: a powerful marker of health. Int J Obes (Lond). 2008;32:1–11. doi:10.1038/sj.ijo.0803774.
3. Ruiz JR, Ortega FB, Gutierrez A, Meusel D, Sjöström M, Castillo MJ. Health-related fitness assessment in childhood and adolescence: a European approach based on the AVENA, EYHS and HELENA studies. J Public Health. 2006;14:269–77. doi:10.1007/s10389-006-0059-z.
4. Dwyer T, Magnussen CG, Schmidt MD, Ukoumunne OC, Ponsonby A-L, Raitakari OT, et al. Decline in physical fitness from childhood to adulthood associated with increased obesity and insulin resistance in adults. Diabetes Care. 2009;32:683–7. doi:10.2337/dc08-1638.
5. Stodden DF, Goodway JD, Langendorfer SJ, Roberton MA, Rudisill ME, Garcia C, Garcia LE. A Developmental Perspective on the Role of Motor Skill Competence in Physical Activity: An Emergent Relationship. Quest. 2008;60:290–306. doi:10.1080/00336297.2008.10483582.
6. Clark JE, Humphrey JH, editors. Motor development: Research and Reviews. 2nd ed. Reston,VA; 2002.
7. Cliff DP, Hesketh KD, Vella SA, Hinkley T, Tsiros MD, Ridgers ND, et al. Objectively measured sedentary behaviour and health and development in children and adolescents: systematic review and meta-analysis. Obes Rev. 2016;17:330–44. doi:10.1111/obr.12371.
8. Jaakkola T, Yli-Piipari S, Huotari P, Watt A, Liukkonen J. Fundamental movement skills and physical fitness as predictors of physical activity: A 6-year follow-up study. Scand J Med Sci Sports. 2016;26:74–81. doi:10.1111/sms.12407.
9. Lubans DR, Morgan PJ, Cliff DP, Barnett LM, Okely AD. Fundamental movement skills in children and adolescents: review of associated health benefits. Sports Med. 2010;40:1019–35. doi:10.2165/11536850-000000000-00000.
10. Larsen LR, Kristensen PL, Junge T, Rexen CT, Wedderkopp N. Motor Performance as Predictor of Physical Activity in Children: The CHAMPS Study-DK. Med Sci Sports Exerc. 2015;47:1849–56. doi:10.1249/MSS.0000000000000604.
11. Bös K. Handbuch sportmotorischer Tests. Göttingen: Hogrefe; 1987.
12. Lämmle L, Tittlbach S, Oberger J, Worth A, Bös K. A Two-level Model of Motor Performance Ability. J Exerc Sci Fit. 2010;8:41–9. doi:10.1016/S1728-869X(10)60006-8.
13. Caspersen CJ, Powell KE, Christenson GM. Physical activity, exercise, and physical fitness: definitions and distinctions for health-related research. Public Health Rep. 1985;100:126–31.
14. Tomkinson GR, Olds TS. Secular Changes in Pediatric Aerobic Fitness Test Performance: The Global Picture. In: Tomkinson GR, Olds TS, editors. Pediatric Fitness: Secular Trends and Geographic Variability. Basel: Karger; 2007. p. 46–66.
15. Greier K, Riechelmann H, Ruedl G, Drenowatz C. Changes in motor competence over four decades in 10 to 14-year-old Austrian boys. CISS 2019. doi:10.15203/CISS_2019.012.

16. Hanssen-Doose A, Niessner C, Oriwol D, Bös K, Woll A, Worth A. Population-based trends in physical fitness of children and adolescents in Germany, 2003-2017. Eur J Sport Sci. 2020:1–11. doi:10.1080/17461391.2020.1793003.
