# Supplementary material for: Secular Trends in Physical Fitness of Children and Adolescents: A Review of Large-Scale Epidemiological Studies Published after 2006
Source: Int J Environ Res Public Health. 2020 Aug 5;17(16):5671. doi: 10.3390/ijerph17165671 (PMC7459672; doi:10.3390/ijerph17165671)
Supplement: Supplementary file 1 [file ijerph-17-05671-s001.zip › Supplementary Material/S3_Full research strategy.pdf]

Search: **((children OR adolescent* OR youth OR child OR Kinder OR Jugendliche) AND (cohort stud* OR Kohortenstudie* OR survey OR longitudinal OR trend* OR secular OR follow-up)) AND ("Motor performance" OR "motorische Leistungsfähigkeit" OR "physical fitness" OR fitness)** Filters: **English, from 2006 - 2020**

((((((((((("child"[MeSH Terms] OR "child"[All Fields]) OR "children"[All Fields]) OR "child s"[All Fields]) OR "children s"[All Fields]) OR "childrens"[All Fields]) OR "childs"[All Fields]) OR "adolescent*"[All Fields]) OR (((("adolescent"[MeSH Terms] OR "adolescent"[All Fields]) OR "youth"[All Fields]) OR "youths"[All Fields]) OR "youth s"[All Fields])) OR (((((("child"[MeSH Terms] OR "child"[All Fields]) OR "children"[All Fields]) OR "child s"[All Fields]) OR "children s"[All Fields]) OR "childrens"[All Fields]) OR "childs"[All Fields])) OR "Kinder"[All Fields]) OR "Jugendliche"[All Fields]) AND ((((((((((((("cohort studies"[MeSH Terms] OR ("cohort"[All Fields] AND "studies"[All Fields])) OR "cohort studies"[All Fields]) OR "cohort"[All Fields]) OR "cohort s"[All Fields]) OR "cohorte"[All Fields]) OR "cohorts"[All Fields]) AND "stud*"[All Fields]) OR "kohortenstudie*"[All Fields]) OR ((((((("survey s"[All Fields] OR "surveyed"[All Fields]) OR "surveying"[All Fields]) OR "surveys and questionnaires"[MeSH Terms]) OR ("surveys"[All Fields] AND "questionnaires"[All Fields])) OR "surveys and questionnaires"[All Fields]) OR "survey"[All Fields]) OR "surveys"[All Fields])) OR ("longitudinal"[All Fields] OR "longitudinally"[All Fields])) OR "trend*"[All Fields]) OR ((((((("secular"[All Fields] OR "secularism"[MeSH Terms]) OR "secularism"[All Fields]) OR "secularity"[All Fields]) OR "secularization"[All Fields]) OR "secularized"[All Fields]) OR "secularizing"[All Fields]) OR "seculars"[All Fields])) OR "follow-up"[All Fields])) AND ((("Motor performance"[All Fields] OR ("motorische"[All Fields] AND "leistungsfahigkeit"[All Fields])) OR "physical fitness"[All Fields]) OR ("fitness"[All Fields] OR "fitnesses"[All Fields]))

Translations

children: "child"[MeSH Terms] OR "child"[All Fields] OR "children"[All Fields] OR "child's"[All Fields] OR "children's"[All Fields] OR "childrens"[All Fields] OR "childs"[All Fields]

youth: "adolescent"[MeSH Terms] OR "adolescent"[All Fields] OR "youth"[All Fields] OR "youths"[All Fields] OR "youth's"[All Fields]

child: "child"[MeSH Terms] OR "child"[All Fields] OR "children"[All Fields] OR "child's"[All Fields] OR "children's"[All Fields] OR "childrens"[All Fields] OR "childs"[All Fields]

cohort: "cohort studies"[MeSH Terms] OR ("cohort"[All Fields] AND "studies"[All Fields]) OR "cohort studies"[All Fields] OR "cohort"[All Fields] OR "cohort's"[All Fields] OR "cohorte"[All Fields] OR "cohorts"[All Fields]

survey: "survey's"[All Fields] OR "surveyed"[All Fields] OR "surveying"[All Fields] OR "surveys and questionnaires"[MeSH Terms] OR ("surveys"[All Fields] AND "questionnaires"[All Fields]) OR "surveys and questionnaires"[All Fields] OR "survey"[All Fields] OR "surveys"[All Fields]

longitudinal: "longitudinal"[All Fields] OR "longitudinally"[All Fields]

secular: "secular"[All Fields] OR "secularism"[MeSH Terms] OR "secularism"[All Fields] OR "secularity"[All Fields] OR "secularization"[All Fields] OR "secularized"[All Fields] OR "secularizing"[All Fields] OR "seculars"[All Fields]
fitness: "fitness"[All Fields] OR "fitnesses"[All Fields]
