# Supplementary material for: Secular Trends in Physical Fitness of Children and Adolescents: A Review of Large-Scale Epidemiological Studies Published after 2006
Source: Int J Environ Res Public Health. 2020 Aug 5;17(16):5671. doi: 10.3390/ijerph17165671 (PMC7459672; doi:10.3390/ijerph17165671)
Supplement: Supplementary file 1 [file ijerph-17-05671-s001.zip › Supplementary Material/Table S1_EPHPP Assessment for included studies .pdf]

Table S1: EPHPP Assessment for included studies

		Ao et al. [44]	Bai et al. [40]	Boddy et al. [24]	Cohen et al. [25]	Costa et al. [26]	Dos Santos et al. [45]	Dyrstad et al. [27]	Ekblom et al. [28]	Fraser et al. [42]	Hardy et al. [43]	Moliner-Urdiales et al. [29]	Moraes Ferrari et al. [46]
**A**	**Selection Bias**	**moderate**	**moderate**	**moderate**	**moderate**	**moderate**	**moderate**	**moderate**	**moderate**	**moderate**	**moderate**	**moderate**	**moderate**
Q1	Are the individuals selected to participate in the study likely to be representative of the target population?<br>1 very likely<br>2 somewhat likely<br>3 not likely<br>4 can't tell	2	2	2	2	2	2	2	2	2	1	2	2
Q2	What percentage of selected individuals agreed to participate?<br>1 80-100% agreement<br>2 60-79% agreement<br>3 less than 60% agreement<br>4 not applicable<br>5 can't tell	5	5	5	5	5	5	5	5	5	5	5	5
**B**	**Study Design**	**moderate**	**moderate**	**moderate**	**moderate**	**moderate**	**moderate**	**moderate**	**moderate**	**moderate**	**moderate**	**moderate**	**moderate**
	Indicate the study design<br>1 randomized controlled trial<br>2 controlled clinical trial<br>3 cohort analytic (two group pre and post)<br>4 case-control<br>5 cohort (one group pre and post (before and after))<br>6 interrupted time series<br>7 other specify<br>8 can't tell	6	6	6	6	6	6	6	6	6	6	6	6
**C**	**Confounders**	**~~strong~~moderate**	**moderate**	**strong**	**strong**	**strong**	**strong**	**moderate**	**moderate**	**strong**	**strong**	**moderate**	**strong**
Q1	Were there important differences between groups prior to the intervention?<br>1 yes<br>2 no<br>3 can't tell	1	1	1	1	1	1	1	1	1	1	1	2
Q2	If yes, indicate the percentage for relevant confounders that were controlled (either in the design (e.g. stratification, matching) or analysis)?<br>1 80-100% agreement<br>2 60-79% agreement<br>3 less than 60% agreement<br>4 can't tell	2	2	1	1	1	1	2	2	1	1	2	2
**D**	**Blinding**	**moderate**	**moderate**	**moderate**	**moderate**	**moderate**	**moderate**	**moderate**	**moderate**	**moderate**	**moderate**	**moderate**	**moderate**
Q1	Was (were) the outcome assessor(s) aware of the intervention or exposure status of participants?<br>1 yes<br>2 no<br>3 can't tell	3	3	3	3	3	3	3	3	3	3	3	3

Q2	Were the study participants aware of the research question?<br>1 yes<br>2 no<br>3 can't tell	3	3	3	3	3	3	3	3	3	3	3	3
**E**	**Data collection methods**	**strong**	**strong**	**strong**	**strong**	**strong**	**strong**	**strong**	**strong**	**strong**	**strong**	**strong**	**strong**
Q1	Were data collection tools shown to be valid?<br>1 yes<br>2 no<br>3 can't tell	1	1	1	1	1	1	1	1	1	1	1	1
Q2	Were data collection tools shown to be reliable?<br>1 yes<br>2 no<br>3 can't tell	1	1	1	1	1	1	1	1	1	1	1	1
**F**	**Withdrawals and drop-outs**	**Weak**	**weak**	**weak**	**weak**	**weak**	**weak**	**strong**	**weak**	**weak**	**weak**	**weak**	**weak**
Q1	Were withdrawals and drop-outs reported in terms of numbers and/or reasons per group?<br>1 yes<br>2 no<br>3 can't tell<br>4 not applicable (i.e. one time surveys or interviews)	2	2	2	2	2	2	1	1	2	2	2	2
Q2	Indicate the percentage of participants completing the study. (if the percentage differs by groups, record the lowest).<br>1 80-100% agreement<br>2 60-79% agreement<br>3 less than 60% agreement<br>4 can't tell<br>5 not applicable (i.e. retrospective case-control)	4	4	4	4	4	4	1	3	4	4	4	4
	GLOBAL RATING	**moderate**	**moderate**	**moderate**	**moderate**	**moderate**	**moderate**	**strong**	**moderate**	**moderate**	**moderate**	**moderate**	**moderate**

		Morales-Demori et al. [41]	Müllerova et al. [31]	Palomäki et al. [30]	Roth et al. [32]	Sandercock et al. [33]	Sandercock et al. [34]	Sedlak et al. [35]	Smpokos et al. [36]	Spengler et al. [37]	Tambalis et al. [38]	Tremblay et al. [14]	Venckunas et al. [39]
**A**	**Selection Bias**	**moderate**	**weak**	**moderate**	**moderate**	**moderate**	**strong**	**moderate**	**moderate**	**moderate**	**strong**	**moderate**	**moderate**
Q1	Are the individuals selected to participate in the study likely to be representative of the target population?<br>1 very likely<br>2 somewhat likely<br>3 not likely<br>4 can't tell	2	1	1	2	2	2	2	2	2	2	2	1
Q2	What percentage of selected individuals agreed to participate?<br>1 80-100% agreement<br>2 60-79% agreement<br>3 less than 60% agreement<br>4 not applicable<br>5 can't tell	5	3	5	5	5	1	5	5	5	1	5	5
**B**	**Study Design**	**moderate**	**moderate**	**moderate**	**moderate**	**moderate**	**moderate**	**moderate**	**moderate**	**moderate**	**moderate**	**moderate**	**moderate**
	Indicate the study design<br>1 randomized controlled trial<br>2 controlled clinical trial<br>3 cohort analytic (two group pre and post)<br>4 case-control<br>5 cohort (one group pre and post (before and after))<br>6 interrupted time series<br>7 other specify<br>8 can't tell	6	6	6	6	6	6	6	6	6	6	6	6
**C**	**Confounders**	**strong**	**strong**	**strong**	**moderate**	**strong**	**strong**	**strong**	**strong**	**strong**	**strong**	**strong**	**strong**
Q1	Were there important differences between groups prior to the intervention?<br>1 yes<br>2 no<br>3 can't tell	2	3	1	1	2	2	2	2	1	1	2	2
Q2	If yes, indicate the percentage fo relevant confounders that were controlled (either in the design (e.g. stratification, matching) or analysis)?<br>1 80-100% agreement<br>2 60-79% agreement<br>3 less than 60% agreement<br>4 can't tell	1	1	1	2	2	1	2	1	1	1	2	1
**D**	**Blinding**	**moderate**	**moderate**	**moderate**	**moderate**	**moderate**	**moderate**	**moderate**	**moderate**	**moderate**	**moderate**	**moderate**	**moderate**
Q1	Was (were) the outcome assessor(s) aware of the intervention or exposure status of participants?<br>1 yes<br>2 no<br>3 can't tell	3	3	3	3	3	3	3	3	3	3	3	3

Q2	Were the study participants aware of the research question?<br>1 yes<br>2 no<br>3 can't tell	3	3	3	3	3	3	3	3	3	3	3	1
**E**	**Data collection methods**	**strong**	**strong**	**strong**	**strong**	**strong**	**strong**	**strong**	**strong**	**strong**	**strong**	**strong**	**strong**
Q1	Were data collection tools shown to be valid?<br>1 yes<br>2 no<br>3 can't tell	1	1	1	1	1	1	1	1	1	1	1	1
Q2	Were data collection tools shown to be reliable?<br>1 yes<br>2 no<br>3 can't tell	1	1	1	1	1	1	1	1	1	1	1	1
**F**	**Withdrawals and drop-outs**	**weak**	**weak**	**strong**	**weak**	**weak**	**weak**	**weak**	**weak**	**weak**	**weak**	**weak**	**weak**
Q1	Were withdrawals and drop-outs reported in terms of numbers and/or reasons per group?<br>1 yes<br>2 no<br>3 can't tell<br>4 not applicable (i.e. one time surveys or interviews)	2	2	1	2	2	2	2	2	2	2	2	2
Q2	Indicate the percentage of participants completing the study. (if the percentage differs by groups, record the lowest).<br>1 80-100% agreement<br>2 60-79% agreement<br>3 less than 60% agreement<br>4 can't tell<br>5 not applicable (i.e. retrospective case-control)	4	4	1	4	4	4	4	4	4	4	4	4
	**GLOBAL RATING**	**moderate**	**weak**	**strong**	**moderate**	**moderate**	**moderate**	**moderate**	**moderate**	**moderate**	**moderate**	**moderate**	**moderate**

Summary: 2 strong [27]; [30]

21 moderate: [44]; [40]: [24]; [25]; [26]; [45]; [28]; [42]; [43]; [29]; [46]; [41]; [32; [33]; [34]; [35]; [36]; [37]; [38]; [14]; [39]

1 weak: [31]
